# Electrical Characterization of Thin PEDOT:PSS Films on Alumina and Thiol–Ene Substrates

**DOI:** 10.3390/polym13203519

**Published:** 2021-10-13

**Authors:** Dalius Jucius, Rimantas Gudaitis, Algirdas Lazauskas, Viktoras Grigaliūnas

**Affiliations:** Institute of Materials Science, Kaunas University of Technology, K. Baršausko 59, LT51423 Kaunas, Lithuania; rimantas.gudaitis@ktu.lt (R.G.); algirdas.lazauskas@ktu.edu (A.L.); viktoras.grigaliunas@ktu.lt (V.G.)

**Keywords:** PEDOT:PSS, thiol–ene, alumina ceramics, transparent, electrical resistance, I–V curves, stability, gauge factor, temperature, relative humidity

## Abstract

Transparent polymer layers that heal minor scratches and maintain the optical properties of the devices for a long time are highly desirable in optoelectronics. This paper presents the results of the electrical characterization of thin PEDOT:PSS films on the novel, optically transparent thiol–ene substrates capable of healing scratches under room-temperature conditions. Electrical properties of the PEDOT:PSS films deposited on the conventional alumina ceramic substrates were also tested for comparative purposes. This study demonstrated that the substrate can have a significant effect on the electrical properties of PEDOT:PSS films, and the electrical resistance of the films on thiol–ene substrates is not as stable as on alumina ceramics. However, the changes in electrical resistance of the films on thiol–ene are small enough over a sufficiently wide range of operating temperatures and relative humidities and allow the application of such bilayers in various polymeric optoelectronic devices.

## 1. Introduction

Current high-performance electronic devices are mostly based on single-crystalline inorganic semiconductors built on rigid substrates. However, in recent decades, innovations in new organic materials and fabrication techniques have led to the rapid development of polymeric semiconductors and conductors essential for next-generation organic electronics [1,2]. The advantages of polymeric materials include their light weight, flexibility, sustainability, biocompatibility, low cost, and the possibility to modify the structure and to tune the properties in a wide range. Polymers can be processed at relatively low temperatures and microstructured using a roll-to-roll compatible time- and cost-efficient hot embossing technique [3]. The solubility of polymers allows the manufacturing of devices by an inexpensive and highly efficient solution processing, including UV imprint and ink-jet printing [4,5]. Numerous examples of successful applications of innovative functional polymers can be found in various fields of electronics, optoelectronics, and photonics and include organic photovoltaic devices, light-emitting diodes, thin-film transistors, displays, touchscreens, nonlinear optical devices, and wearable and automotive electronics [1,5,6,7]. In addition, various types of polymer-based sensors have been continuously developed for applications in healthcare, environmental monitoring, control of chemical reactions, and the emerging Internet of Things [8,9,10].

Poly(3,4-ethylenedioxythiophene):poly(styrenesulfonate) (PEDOT:PSS) is the most commonly used commercially available conducting polymer for organic electronic devices [11,12,13]. This polymer exhibits many unique properties, such as high optical transparency, excellent thermal stability, solubility in water, physical and chemical stability in air, extremely high flexibility, and good film-forming ability. PEDOT:PSS films consist of conducting PEDOT-rich grains encapsulated by insulating PSS-rich shells and can be easily fabricated by conventional solution processing techniques [14]. One of the main drawbacks of PEDOT:PSS is associated with its relatively low inherent conductivity, which is usually below 1 S/cm. To improve conductivity, several methods, such as the addition of high-boiling-point polar organic solvents, ionic liquids, or surfactants; post-treatment with organic solvents, polar solvent vapor, salts, zwitterions, or acids; and combinations of these, have been explored, resulting in an increase in conductivity by two or three orders of magnitude [15,16,17,18,19,20]. Recently reported results demonstrated that it is possible to enhance the conductivity of PEDOT:PSS films to more than 4000 S/cm [21], which is comparable to that of indium tin oxide (ITO) and allows the PEDOT:PSS films to be used as transparent electrodes of touchscreens and solar cells.

Transparent PEDOT:PSS electrodes are generally coated on polydimethylsiloxane (PDMS), polyethylene terephthalate (PET), polyimide (PI), and polycarbonate (PC) substrates conventionally used for the fabrication of optoelectronic devices [22,23,24,25]. Such substrates are able to provide sufficient mechanical strength and, at the same time, to perform the function of a protective layer. However, accidental cuts and scratches accumulate on the surface over time and the optical properties of these protective layers deteriorate. To overcome this problem, self-healing materials capable of healing minor scratches and maintaining optical properties for a long time would be very promising as protective layers [26,27]. Particularly attractive are intrinsic self-healing materials that are able to heal multiple times and do not contain microcapsules or vascular networks that alter optical transmittance [28].

However, the electrical properties of PEDOT:PSS thin films are highly dependent on the processing methods, environmental factors, such as temperature and humidity, applied stresses, and peculiarities of the substrate [29,30,31]. It has been proposed to use dimethyl sulfoxide (DMSO) additives, ethylene glycol (EG), or strong acid treatment in order to increase the stability of the films [32,33,34]. The influence of substrate roughness has also been examined [35]. However, the electrical properties of PEDOT:PSS films fabricated on self-healing substrates have not been studied so far.

In a previous work, we fabricated and tested highly optically transparent thiol–ene coatings capable of healing scratches under room-temperature conditions [36]. These coatings can be permanently microstructured by UV casting or hot embossing, and are capable of recovering mechanically deformed surface microstructures after heat treatment above the glass transition temperature of the polymer [37,38]. Recently, we reported on the fabrication of PEDOT:PSS and thiol–ene bilayers for optoelectronic applications, and described the initial results on the measurements of the mechanical and optical properties of such composite films [39]. This paper presents the results of the electrical characterization of DMSO-doped PEDOT:PSS films on thiol–ene substrates. Electrical properties of the same conductive polymer films on the conventional in-microelectronics alumina ceramic substrates were also tested for comparison.

## 2. Materials and Methods

### 2.1. Materials

1,3,5-triallyl-1,3,5-triazine-2,4,6(1H,3H,5H)-trione (TTT, trifunctional allyl component), pentaerythritol tetrakis(3-mercaptopropionate) (PETMP, tetrafunctional thiol component), and 2,2-dimethoxy-2-phenylacetophenone (DMPA, photoinitiator) were purchased from Sigma-Aldrich (St. Louis, MO, USA). PEDOT:PSS (doped with 5% DMSO, 1.1 wt% solution in water) was purchased from PEDOT Inks LLC (Cedar Park, TX, USA). The two-side polished alumina ceramic substrates (Al_2_O_3_) were purchased from MTI Corporation (Richmond, CA, USA).

### 2.2. Fabrication of Samples

The photopolymerizable thiol–ene composition was prepared as a mixture of PETMP and TTT with a 1:1 stoichiometric ratio of thiol to ene functional groups, containing 1 wt% of DMPA. Details of the preparation procedure have been reported previously [36]. The clear, colorless, viscous mixture of PETMP and TTT was applied on polytetrafluoroethylene (PTFE) plates as a 150 ± 1 µm-thick layer via the Meyer rod coating method. Some samples were prepared on alumina substrates. All samples were UV-cured simultaneously at intensities of 1.64 mW/cm^2^ (254 nm wavelength) and 0.8 mW/cm^2^ (365 nm wavelength) for 5 min. The PEDOT:PSS dispersion was spin-coated on the alumina and thiol–ene at 2000 rpm for 30 s. Subsequently, the samples were dried at room temperature and cured on a hot plate at 100 °C for 10 min. Prior to the thiol–ene application and spin-coating procedure, the alumina and thiol–ene surfaces were exposed to O_2_ radio-frequency (RF) plasma in the camera of the device Plasma-600-T (JSC Kvartz, Kaliningrad, Russia) at 133 Pa of pressure (RF = 13.56 MHz, *p* = 0.3 W/cm^2^, t = 30 s) in order to improve wetting characteristics. Free-standing PEDOT:PSS on thiol–ene films was obtained by gently peeling the film from the PTFE plate. Therefore, three types of specimens were prepared: (1) PEDOT:PSS on alumina substrate, (2) PEDOT:PSS on thiol–ene substrate, and (3) PEDOT:PSS on thiol–ene-coated alumina substrate. Type 3 was used only for piezoresistive testing.

### 2.3. Characterization

Contact angle (CA) measurements were performed at room temperature using the sessile drop method. A droplet of ultrapure water (5 μL) was deposited on the investigated surface. Optical images of the droplet were recorded with a PC-connected digital camera after 10 s from the dropping, and CA measurements were carried out using an active contour method based on B-spline snakes (active contours) [40].

Atomic force microscopy (AFM) experiments were carried out at room temperature using a NanoWizardIII atomic force microscope (JPK Instruments, Bruker Nano GmbH, Berlin, Germany), while the data were analyzed using SurfaceXplorer and JPKSPM Data Processing software (Version spm-4.3.13, JPK Instruments, Bruker Nano GmbH, Berlin, Germany). The AFM images were collected using an ACTA (Applied NanoStructures, Inc., Mountain View, CA, USA) probe (tip shape—pyramidal, radius of curvature (ROC) < 10.0 nm, and cone angle—20°; silicon cantilever shape—pyramidal, reflex side coating—Al with thickness of 50 ± 5 nm, calibrated spring constant—54.2 N/m, and set point—195.48 nN) operating in contact mode.

**Figure 1:**
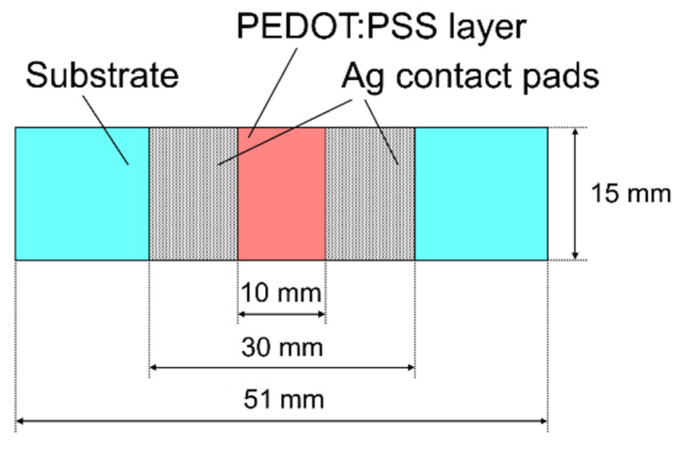
Layout of tested samples.

The layout of the samples used for electrical characterization of the PEDOT:PSS films is shown in Figure 1. The dimensions of the substrates were 15 × 51 mm. Rectangular contact pads were formed on the top of the PEDOT:PSS layer using a commercial Ag paste to ensure good electrical contact. The distance between the contact pads was equal to 10 mm.

A Keitley 6487 picoammeter (Keithley Instruments Inc., Cleveland, OH, USA) was used to record the current–voltage (I–V) characteristics. The resistance of the samples was calculated from the measured I–V curves. The results of the measurements of five samples were averaged before further analysis.

**Figure 2:**
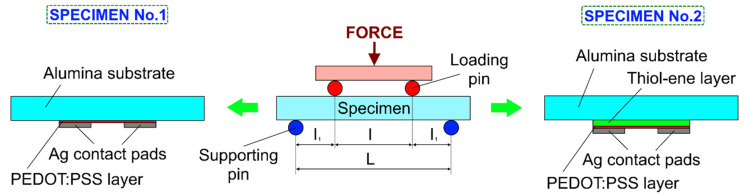
Four-point bending test setup.

The piezoresistive properties of PEDOT:PSS films were evaluated using a four-point bending method (Figure 2). This method was chosen because it provides a constant bending moment between the two loading pins. In this case, the relative elongation (tensile strain) of the sample Δ*l*/*l* is related to the applied force *F* by the following equation [41]:

(1) $$\frac{\Delta l}{l}=\frac{3F\left(L-l\right)}{2bh^{2}E},$$

where *l* is the distance between loading pins; *L* is the distance between supporting pins; *b*, *h*, and *E* are the width, thickness, and Young‘s modulus of the sample, respectively. In our case, the variables’ listed values of the above-listed variables were as follows: *l* = 24 mm, *L* = 42 mm, *b* = 15 mm, *h* = 0.5 mm, and *E* = 350 GPa.

The temperature dependence of resistance was measured by placing the sample on a Peltier element and changing its temperature from 15 to 70 °C with a temperature increase rate of about 5.5 °C/min. The dependence of resistance on relative humidity was estimated using a custom-made measurement setup (KTU, Kaunas, Lithuania). Relative humidity was varied from 30 to 90% with an increase rate of approximately 5%/min. The long-term stability of the samples was evaluated by storing them for 30 days under room-temperature conditions.

## 3. Results and Discussion

Contact angle measurements showed that the surface wettabilities of the as-received alumina substrates and as-prepared thiol–ene films were poor. To strengthen the adhesion between the substrates and the spin-coated PEDOT:PSS films and to prevent possible dewetting problems, O_2_ plasma treatment of the surfaces was carried out. The plasma treatment performed was very efficient and the water contact angles on the surfaces of alumina and thiol–ene decreased from 89 ± 2° and 87 ± 2° to 10 ± 2° and 16 ± 3°, respectively, resulting in a highly hydrophilic nature of the treated surfaces.

**Figure 3:**
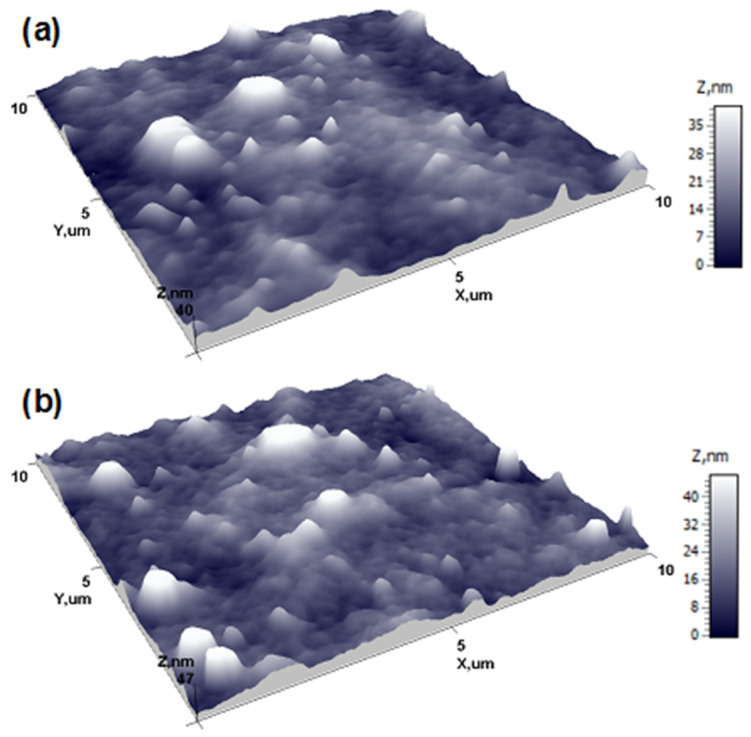
AFM surface topography of PEDOT:PSS film on alumina (**a**) and thiol–ene (**b**) substrates.

After the spin-coating procedure, fairly smooth, defect-free PEDOT:PSS films were formed on the plasma-treated surfaces. AFM analysis of the films coated on alumina and thiol–ene substrates.(Figure 3a,b) was carried out over a 10.0 × 10.0 µm^2^ area for quantitative morphological evaluation. The surface topography of the PEDOT:PSS film on the alumina substrate exhibited a random distribution of surface features that had different angular orientations from each other, without a preferred direction. The mean height of the surface structures (*Z*_mean_) was determined to be 14.31 nm. The root-mean-square roughness (*R*_q_) was found to be 6.35 nm. The surface peaks dominated over the valleys in the height distribution with a skewness (*R*_sk_) value of 1.49 and exhibited a leptokurtic distribution of the morphological features with a kurtosis (*R*_ku_) value of 6.42. The *Z*_mean_ and *R*_q_ values for PEDOT:PSS on the thiol–ene substrate were found to be 16.98 nm and 7.44 nm, respectively. The topography followed a similar distribution of surface morphological features with *R*_sk_ and *R*_ku_ values of 1.67 and 6.77, respectively. The thickness of the PEDOT:PSS films was determined from scratched AFM samples: 87 ± 7 nm and 71±8 nm for the samples on alumina and thiol–ene substrates, respectively.

**Figure 4:**
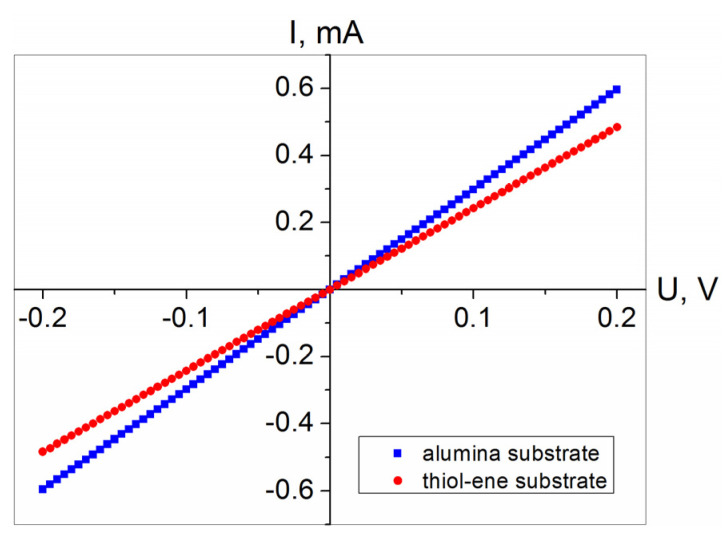
Characteristic current–voltage curves of PEDOT:PSS films on alumina and thiol–ene substrates.

**Figure 5:**
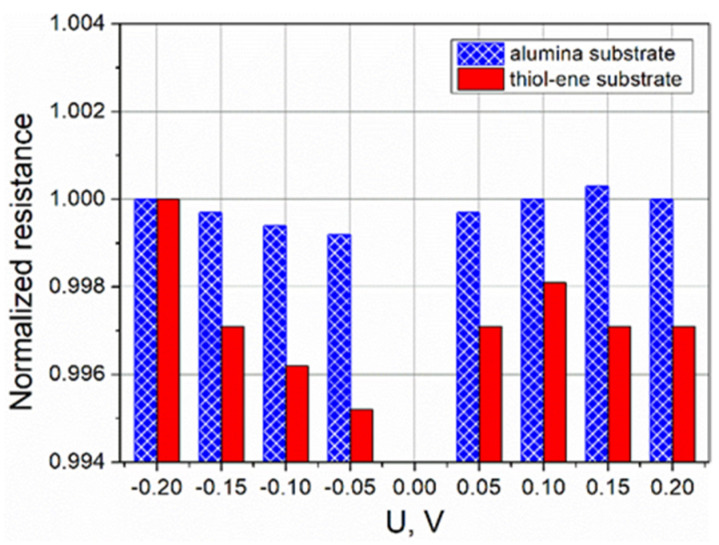
Dependence of resistance on applied voltage for PEDOT:PSS films on alumina and thiol–ene substrates. Histograms are normalized with respect to resistance at −0.2 V.

In the first stage of electrical characterization of the fabricated PEDOT:PSS films, I–V curves were obtained by increasing the applied voltage from −0.2 to +0.2 V in 5 mV increments at intervals of 0.2 s. Typical examples of the I–V curves obtained for the films coated on the alumina and thiol–ene substrates are presented in Figure 4. In both cases, the I–V curves exhibited an ohmic behavior, i.e., the concentration and mobility of charge carriers in the films were independent of the applied voltage, and the electrical current was directly proportional to the voltage applied across the film. However, the resistance of the films on alumina substrate was lower. The main reason for this difference should be related to the effect of substrate surface energy on the thickness of the coated PEDOT:PSS film. During voltage stepping, the resistance was very stable with a variation ratio Δ*R*/*R*_min_ below 0.1% for samples on alumina substrates and less than 0.5% for samples on thiol–ene substrates (*R*_min_ stands for minimal resistance in the voltage range mentioned above). The exact causes of such a difference are not fully understood, but the possible reason for this might be related to the differences in the PEDOT:PSS film-forming ability on the tested substrates. Histograms illustrating the measured resistance change with applied voltage for the PEDOT:PSS films on alumina and thiol–ene substrates are displayed in Figure 5.

**Figure 6:**
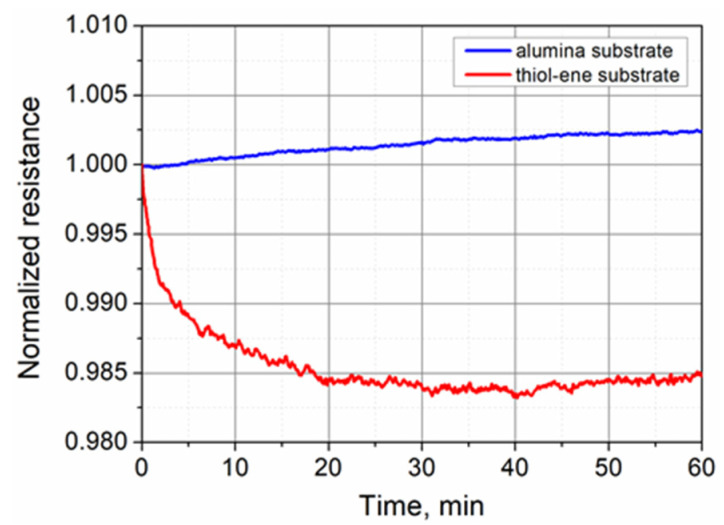
Normalized electrical resistance of PEDOT:PSS films on alumina and thiol–ene substrates versus time over a 60 min period.

In general, PEDOT:PSS, especially doped with DMSO, has been reported to be more stable compared to other conducting polymers [42]. However, the irreversible increase in its electrical resistance with time when exposed to air atmosphere may still substantially limit the range of possible applications in electronics. To evaluate the stability of the fabricated PEDOT:PSS films under room-temperature conditions, we performed short-term and long-term stability tests in air ambient. For short-term stability testing, the electrical resistance of the films was measured every 2 s over a period of 60 min at 0.1 V of applied voltage. The results of the measurements are presented in Figure 6. It is evident that the electrical resistance of the PEDOT:PSS films on the alumina substrate remained almost stable over one hour. During this period, the slow increase in resistance did not exceed 0.25%. Thiol–ene is an inferior heat conductor compared to alumina. As a result, the films formed on the thiol–ene substrate became hotter due to the Joule effect as electrical current flowed through them. The decrease in resistance of these films was fastest in the first 10 min, when the change reached 1.31%. Thereafter, as a result of heat exchange with the ambient, the temperature of the PEDOT:PSS layer reached the steady-state condition, while the resistance stabilized and even slightly increased during the last 20 min of the measurements as a consequence of the interaction with the environment. Based on the results of the measurements performed, it can be stated that under the steady-state condition, the current flow in the PEDOT:PSS film on the thiol–ene substrate caused a 1.68% decrease in resistance. If we consider the temperature coefficient of resistance (TCR) for PEDOT:PSS to be equal to ~−0.25%/°C [43], a recorded 1.68% decrease in resistance corresponds to about a 6.7–7.0 °C increase in the film temperature. From 30 to 60 min of the measurements, the resistance change of PEDOT:PSS films on the thiol–ene substrate did not exceed +0.12% and was close to +0.09% recorded for the samples on the alumina substrate.

**Figure 7:**
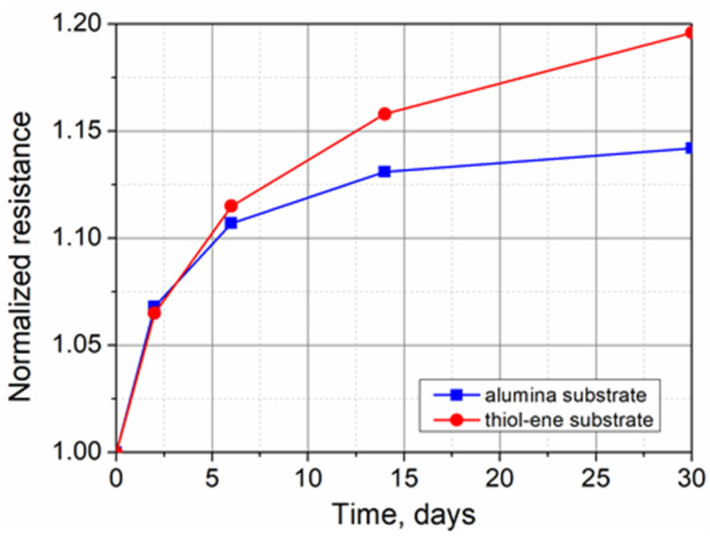
Evolution of the electrical resistance of PEDOT:PSS films on alumina and thiol–ene substrates with time over a period of 1 month.

Long-term stability was evaluated by measuring the electrical resistance of the PEDOT:PSS films immediately after spin-coating, and after 2, 6, 14, and 30 days of storage under room-temperature conditions. The evolution of resistance with time over a period of 1 month is shown in Figure 7. The observed increase in resistance of the tested samples can be explained by the oxidation of conjugated molecules under the influence of oxygen and water vapor absorption from the surrounding environment. The resistance changes were most pronounced in the first 2 days, reaching up to 7% of the initial value. Later, the resistance gradually stabilized. From day 14 to day 30, the changes in resistance of PEDOT:PSS films on alumina and thiol–ene substrates were only 0.97% and 3.28%, respectively. Thus, in both cases, sufficient electrical stability of PEDOT:PSS films in the ambient atmosphere was obtained.

**Figure 8:**
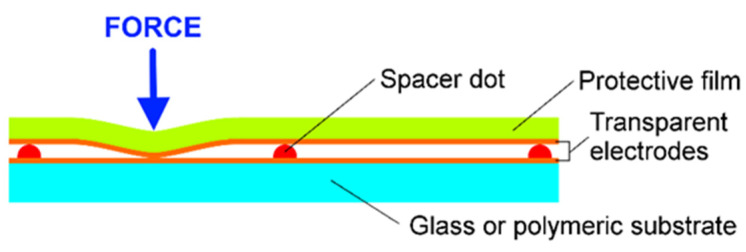
Design of a resistive touch screen.

A four-point bending method was used to evaluate the piezoresistive properties of the fabricated PEDOT:PSS films. Using this method, the relative elongation of a sample between the two loading points under a fixed applied force *F* is described by Equation (1). The most common resistive touchscreens (Figure 8) consist of two transparent sheets coated with a thin layer of conducting material and slightly separated by spacer dots. When a sufficient force is applied to the upper sheet, electrical contact between the conductive films is established and the position of the touch can be determined. Usually, spacer dots have heights of at least ~2 µm, height-to-diameter aspect ratios of at least ~1:10, and spacings of the spacer dots of at least ten times the diameter [44,45]. As a result, only a small relative elongation of the upper sheet is required to obtain electrical contact of the electrodes. If the height of the spacer dots is 10 µm and the distance between the dots is 1 mm, then the minimum relative elongation of the upper sheet necessary to ensure electrical contact is equal to 0.02%. This elongation in our case corresponds to a load of 1 kg or an applied force of 9.72 N. Taking this into account, we periodically varied the applied load between 0 and 1.4 kg in 50 g steps, and the applied force changed from 0 to 13.73 N. A full load–unload cycle lasted 114.8 s.

**Figure 9:**
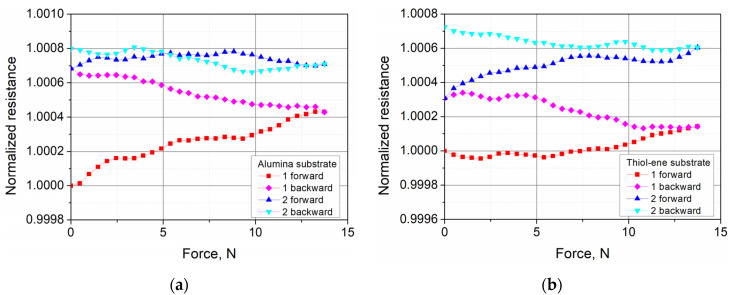
Dependence of the normalized resistance of PEDOT:PSS films on the applied force during the first two cycles of loading: (**a**) alumina substrate and (**b**) thiol–ene substrate on alumina. The notation “forward” means an increase in force, while “backward” means a decrease.

The evolution of the normalized resistance of the PEDOT:PSS films during the first two loading cycles is presented in Figure 9. It is interesting to note that, regardless of the substrate used, the resistance of the investigated films increased all the time with the increase and decrease in the load. The observed increase, regardless of the phase of load–unload cycle, is related to the irreversible rearrangement of the polymer chains under periodic loading. When PEDOT:PSS films were coated directly on the alumina substrate, their resistance increased fastest during the first cycle. Meanwhile, for the films coated on the thiol–ene layer, the changes in resistance were similar over both cycles.

**Figure 10:**
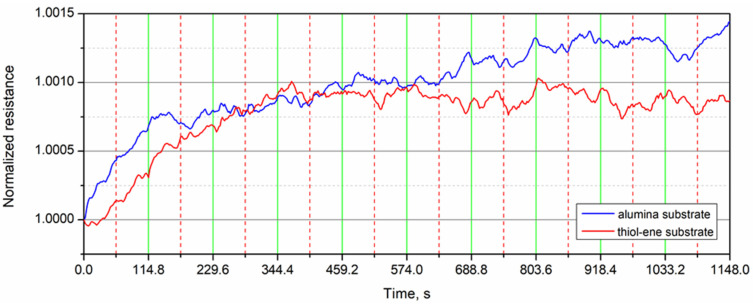
Normalized resistance of the PEDOT:PSS films versus time over 10 cycles of applied strain. The vertical dashed red lines correspond to the maximum load and the solid green lines correspond to zero load.

A more complete picture of the changes that occur can be obtained by analyzing the time dependencies of the normalized resistance over 10 cycles of applied strain presented in Figure 10. From this figure, it is evident that the resistance of the samples on alumina increased all the time, but the fastest increase was recorded during the first cycle. Meanwhile, for the samples on thiol–ene, the fastest increase in resistance was obtained during the first three cycles, after which the resistance stabilized. Such differences in the character of the curves can be explained by the effect of two factors: the higher Joule heating of the PEDOT:PSS films on thiol–ene and the damping effect of the thiol–ene layer, which reduces the tensile strain of the PEDOT:PSS film under certain loads of the sample. In general, the changes in resistance of PEDOT:PSS films tested after 10 load–unload cycles were very small and did not exceed +0.14% for the films coated on alumina and +0.07% for the films on thiol–ene. The dependence of the resistance on the applied force was not very clear.

**Figure 11:**
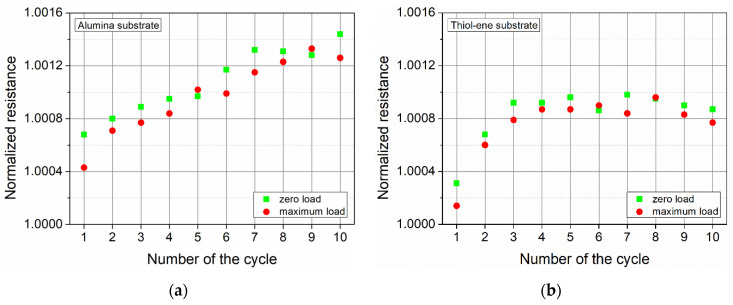
Normalized resistance of the PEDOT:PSS films at zero and maximum load versus the number of load–unload cycles: (**a**) alumina substrate and (**b**) thiol–ene substrate on alumina.

To highlight the changes in resistance due to the applied force, Figure 11 presents the dependence of the resistance of PEDOT:PSS films at zero and maximum load on the number of load–unload cycles. The data show that in most cases, tensile strain reduced the resistance of PEDOT:PSS films. The effect of the applied tensile strain on the resistance of the tested films was quantitatively evaluated by calculating the gauge factor (GF):

(2) $$GF=\frac{\left(R_{1}-R_{0}\right)/R_{0}}{\Delta l/l}\;$$

where *R*_0_ is the resistance at zero load, *R*_1_ is the resistance at maximal load, and ∆*l*/*l* is the tensile strain.

During the first two cycles of loading, the average GF for the films on alumina and thiol–ene was equal to 0.11 and 0.16, respectively. On the other hand, when the rapid change in resistance process was already over, i.e., during the 3–10 cycles, the average GF for the PEDOT:PSS films became negative and was equal to −0.19, regardless of the substrate used. This result is the superposition of geometrical and piezoresistive effects partially cancelling each other [31]. The geometric component of GF is predetermined by the Poisson’s ratio ν and can be expressed as 1 + 2ν. The Poisson’s ratio of PEDOT:PSS nanofilms is equal to 0.33 [46]. Thus, the geometrical component of GF for such films can be estimated to be equal to 1.66. Thus, it can be concluded that the negative piezoresistive component of GF equal to −1.85 is dominant in our films regardless of the substrate used. The origin of this component can be attributed to the alignment of conjugated polymer chains in the direction of applied strain, resulting in a decrease in resistivity.

**Figure 12:**
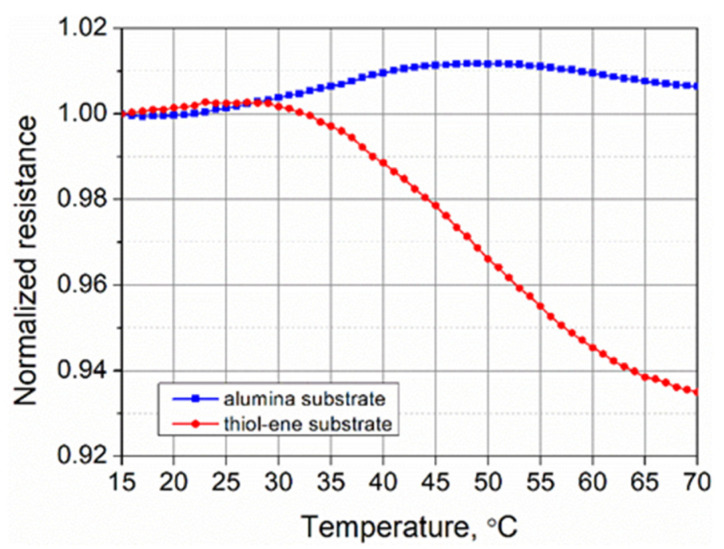
Normalized resistance of PEDOT:PSS films on alumina and thiol–ene substrates versus temperature.

The influence of temperature on the electrical conductivity of PEDOT:PSS films was evaluated by measuring their resistance in the temperature range of 15–70 °C. The results of the measurements are shown in Figure 12. The transport of charge carriers in PEDOT:PSS occurs by thermally assisted hopping among localized states at different energies [47,48]. The characteristic hopping length is a function of the distance between two localized states and the difference in associated energies. The thermal vibrations of the polymer chains increase the difference in energy of these states. Thus, it would be likely to expect that heating of the samples would result in a decrease in their resistance because of the decrease in the optimal hopping distance. However, in our case, the dependence of the resistance on temperature had a more complex character, which was determined by the type of substrate used. We believe that this could be related to the different coefficients of thermal expansion (CTEs) of the PEDOT:PSS films and substrates, resulting in the deformation of the conducting layer. CTEs have been reported to be approximately 53 × 10^−6^/°C for PEDOT:PSS films [49], 8 × 10^−6^/°C for alumina ceramics [50], and 151 × 10^−6^/°C for thiol–ene [51]. Thus, the influence of alumina and thiol–ene substrates on PEDOT:PSS films is the opposite. All our samples were fabricated at 20 °C. Near this temperature, the resistance of the samples changed very slightly. Above 25–30 °C, the changes were more significant. The alumina substrate began to hinder the thermal expansion of the PEDOT:PSS layer. The compressive strain experienced reordered the PEDOT:PSS chains, thus increasing the resistance of the conducting layer. At the same time, an increase in electrical conductivity of PEDOT:PSS was observed due to the thermally assisted hopping process, but compressive strain was the dominant factor causing the resistance of the sample to increase. As the samples on alumina were heated further, the influence of chain reordering decreased and the increase in electrical conductivity began to dominate above 49 °C. For the samples on thiol–ene, above room temperature, the substrate began to stretch the PEDOT:PSS film, thus reducing its resistance. At the same time, the resistance decreased due to the increase in electrical conductivity associated with the decrease in characteristic hoping distance. Both factors, acting in the same direction, led to a larger decrease in resistance compared to the samples on the alumina substrate. Based on the results presented in Figure 12, the maximum relative change in resistance of PEDOT:PSS films tested in the temperature range of 15–70 °C was calculated to be 1.18% and 6.51% for the samples on alumina and thiol–ene substrates, respectively. The TCR value for PEDOT:PSS films on the thiol–ene substrate at 50 °C was estimated to be approximately −0.24%/°C.

**Figure 13:**
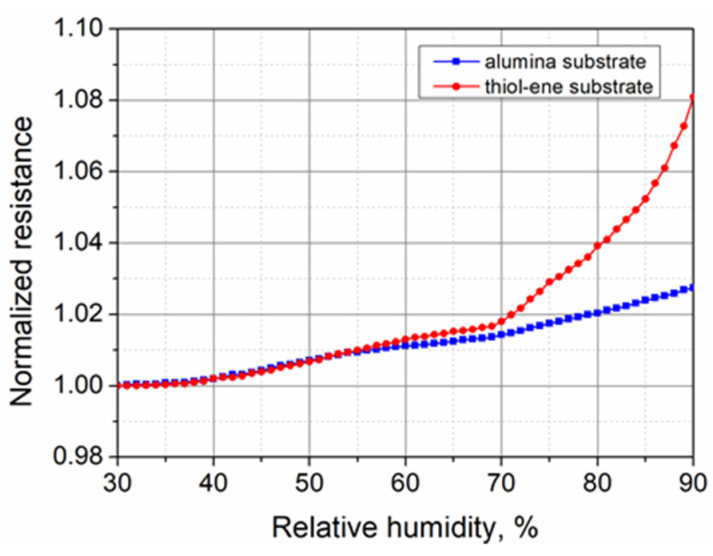
Normalized resistance of PEDOT:PSS films on alumina and thiol–ene substrates versus relative humidity.

It is known that due to the hydrophilic nature of polystyrene sulfonate, PEDOT:PSS can readily absorb water from the surrounding environment. Therefore, in the last stage of the research, we measured the relative humidity dependence of the PEDOT:PSS resistance. The results of the measurements in the range of relative humidity between 30 and 90% are shown in Figure 13. It can be seen that with increasing relative humidity, the resistance of PEDOT:PSS films increased on both substrates. The increase in the resistance of the samples on the alumina substrate was nearly constant and equal to about 0.04%/%RH over the entire range of relative humidity. This increase in resistance can be attributed to the adsorption and diffusion of water vapor throughout the bulk of the film, causing swelling of the PSS-rich matrix and the separation of the conducting PEDOT-rich domains, and thus making the electron hopping process more difficult [13,52]. For the samples on the thiol–ene substrate, the increase in resistance in the range from 30 to 70% relative humidity was very similar to that observed for the samples on the alumina substrate. However, above a relative humidity of 70%, the rate of the change in resistance increased significantly and reached up to 0.31%/%RH. The main reason for this can be explained by swelling of the thiol–ene substrate at higher relative humidity due to the polarity and affinity to water of the ester groups present in the thiol crosslinker (PETMP) [53]. The total increase in resistance in the range from 30 to 90% of the relative humidity did not exceed 2.66% and 8.10% for the samples on alumina and thiol–ene substrates, respectively. Thus, the performed experiments demonstrated sufficient stability of the electrical resistance of the PEDOT:PSS films over a wide range of relative humidity, with the exception of the films on the thiol–ene substrate at above 75–80% of the relative humidity.

## 4. Conclusions

The purpose of this study was to perform an electrical characterization of thin PEDOT:PSS films on the novel, optically transparent thiol–ene substrates capable of healing scratches under room-temperature conditions, and to compare them with the same films on conventional in-microelectronics alumina ceramic substrates. The short O_2_ plasma treatment of substrates was found to be very effective in enhancing surface wettability and preventing problems of dewetting during the spin-coating of PEDOT:PSS. Fairly smooth and defect-free PEDOT:PSS films with linear I–V characteristics were coated on the plasma-treated surfaces, though some difference in the film thickness related to the effect of substrate surface energy was observed. Short-term stability tests demonstrated noticeable Joule heating of the films on thiol–ene substrates, causing a decrease in resistance by 1.7% during the first 20 min. Later, the steady-state condition of heat transfer was achieved and the further change in the resistance did not exceed +0.12%, which was similar to the +0.09% observed for the films on alumina. Testing of the long-term stability showed that the fastest changes in resistance due to the interaction of the films with the environment occurred during the first 2 days. Afterward, the resistance gradually stabilized, and from day 14 to day 30, the changes did not exceed 0.97% and 3.28% for the films on alumina and thiol–ene substrates, respectively, indicating sufficient electrical stability of the films. The piezoresistance of the tested PEDOT:PSS films was small and, after the first load cycles, the piezoresistive component of the gauge factor was equal to −1.85 regardless of the substrate used. The dependence of the resistance on temperature was significantly influenced by differences in the thermal expansion coefficients of the PEDOT:PSS films and the substrates. The maximum relative resistance change in the temperature range of 15–70 °C was calculated to be 1.18% and 6.51% for the samples on alumina and thiol–ene, respectively. The evaluation of the influence of relative humidity in the range of 30–90% RH showed that the resistance change rate was about 0.04%/%RH, except for the samples on the thiol–ene substrate above 70% RH, where the substrate began to swell and the rate increased to 0.31%/%RH.

Therefore, the main conclusion of this research is that the substrate can have a significant effect on the electrical properties of PEDOT:PSS films, and the electrical resistance of the films on the thiol–ene substrate is not as stable as that on alumina ceramics. The main reason for this is the larger coefficient of thermal expansion, worse heat dissipation, swelling at high relative humidity, and natural ageing of the thiol–ene substrates used. However, the changes in electrical resistance of the films on thiol–ene are small enough over a sufficiently wide range of operating temperatures and relative humidities, and allow the application of such bilayers in various polymeric optoelectronic devices where the ability of the protective layer to heal scratches is desirable.

## Data Availability

The data of this study will be available from the authors upon request.
